# An Open View on SARS-CoV-2 Infection

**DOI:** 10.3390/microorganisms12010156

**Published:** 2024-01-12

**Authors:** Erica Diani, Davide Gibellini

**Affiliations:** Microbiology Section, Department of Diagnostic and Public Health, University of Verona, 37134 Verona, Italy; davide.gibellini@univr.it

The onset of the SARS-CoV-2 virus led to the appearance of a devastating pandemic, which once again demonstrated the practical importance of virology.

There have been massive investments in studies on the pathogenesis mechanisms of the SARS-CoV-2 infection, including the development of diagnostic methods, vaccines and antiviral therapies which can counteract the infection, especially in its most severe clinical forms, or can prevent SARS-CoV-2 infection in a very short timeframe. The use of advanced technologies including NGS methods has enabled the monitoring of viral mutations, the description of viral genome evolution and the comprehension of several aspects of virus/host interaction dynamics during the progressive waves of the virus. The use of these methods has played an essential role in shedding light on the characteristics of the virus’ biology with respect to its consistency of replication in target cells and its ability to induce clinical manifestations by coupling clinical observations with the genome characteristics of different viral lineages. Furthermore, the application of RNA vaccines generated a series of very functional vaccines that successfully tackled the spread of the viral infection and its severe clinical manifestations. In addition to these observations, many studies have also displayed the pivotal importance of the immunological aspects and the role of comorbidities in the development of COVID-19’s manifestations.

In this Special Issue, we would like offer to the reader an extensive vision on the world of SARS-CoV-2, with studies on molecular mimicry as the mechanism underlying autoimmune attacks on the CNS [1], on the prevalence of invasive fungal infections in SARS-CoV-2-infected patients [2], on the unpredictable dynamics of the antibody response in vaccinated and naturally infected patients [3], on ORF8 deletion mutants detected via NGS screening [4], and on the induction of liver damage by SARS-CoV-2 [5]. These different aspects are very useful for recognizing the complexity of SARS-CoV-2 infection, demonstrating that it is not only a respiratory infection, but rather that it plays a multifaceted role affecting several organs and tissues.
